# Laparoscopic pancreatectomy for benign or low-grade malignant pancreatic tumors: outcomes in a single high-volume institution

**DOI:** 10.1186/s12893-021-01414-w

**Published:** 2021-12-07

**Authors:** He Cai, Lu Feng, Bing Peng

**Affiliations:** 1 Department of Pancreatic Surgery, West China Hospital, Sichuan University, No. 37, Guo Xue Xiang, Chengdu, 610041 Sichuan China; 2 Department of Minimal Invasive Surgery, Shangjin Nanfu Hosptial, Chengdu, China; 3 Operating Room of Anesthesia Surgery Center, West China Hospital/West China School of Nursing, Sichuan University, Chengdu, China

**Keywords:** Laparoscope, Pancreatectomy, Outcomes, Benign and low-grade malignant pancreatic tumors, Surgical techniques

## Abstract

**Objective:**

To investigate the perioperative and long-term outcomes of laparoscopic pancreatectomy for benign and low-grade malignant pancreatic tumors, and further compare the outcomes between different surgical techniques.

**Methods:**

We retrospectively collected clinical data of consecutive patients with benign or low-grade malignant pancreatic tumors underwent surgery from February 2014 to February 2019. Patients were grouped and compared according to different surgical operations they accepted.

**Results:**

Totally 164 patients were reviewed and 83 patients underwent laparoscopic pylorus-preserving pancreaticoduodenectomy (LPPPD), 41 patients underwent laparoscopic spleen-preserving distal pancreatectomy (LSPDP) and 20 patients underwent laparoscopic central pancreatectomy (LCP) were included in this study, the rest 20 patients underwent laparoscopic enucleation were excluded. There were 53 male patients and 91 female patients. The median age of these patients was 53.0 years (IQR 39.3–63.0 years). The median BMI was 21.5 kg/m^2^ (IQR 19.7–24.0 kg/m^2^). The postoperative severe complication was 4.2% and the 90-days mortality was 0. Compare with LCP group, the LPPPD and LSPDP group had longer operation time (300.4 ± 89.7 vs. 197.5 ± 30.5 min, *P* < 0.001) while LSPDP group had shorter operation time (174.8 ± 46.4 vs. 197.5 ± 30.5 min, *P* = 0.027), more blood loss [140.0 (50.0–1000.0) vs. 50.0 (20.0–200.0) ml *P* < 0.001 and 100.0 (20.0–300.0) vs. 50.0 (20.0–200.0 ml, *P* = 0.039, respectively), lower rate of clinically relevant postoperative pancreatic fistula [3 (3.6%) vs. 8 (40.0%), *P* < 0.001 and 3 (7.3%) vs. 8 (40.0%), *P* = 0.006, respectively], lower rate of postpancreatectomy hemorrhage [0 (0%) vs. 2 (10.0%), *P* = 0.036 and (0%) vs. 2 (10.0%) *P* = 0.104, respectively] and lower rate of postoperative severe complications [2 (2.4%) vs.4 (20.0%), *P* = 0.012 and 0 (0%) vs. 4 (20.0%), *P* = 0.009, respectively], higher proportion of postoperative pancreatin and insulin treatment (pancreatin: 39.8% vs., 15% *P* = 0.037 and 24.4%vs. 15%, *P* = 0.390; insulin: 0 vs. 18.1%, *P* = 0.040 and 0 vs. 12.2%, *P* = 0.041).

**Conclusions:**

Overall, laparoscopic pancreatectomy could be safely performed for benign and low-grade malignant pancreatic tumors while the decision to perform laparoscopic central pancreatectomy should be made carefully for fit patients who can sustain a significant postoperative morbidity and could benefit from the excellent long-term results even in a high-volume center.

## Introduction

Pancreatectomy is the standard treatment for benign and low-grade malignant pancreatic tumors. The first laparoscopic pancreatectomy for islet cell tumors of the pancreas was performed in 1992 [1]. During the following two decades, although many studies have tried to demonstrate the safety and feasibility of laparoscopic pancreatectomy [2–8]. The famous randomized controlled LEOPARD-2 trial was terminated early because of high mortality in the laparoscopic group which indicated the surgeon volume was significantly associated with perioperative outcomes [9–11]. What’s more, the only systematic review and meta-analysis of randomized controlled trials also found that laparoscopic pancreaticoduodenectomy (LPD) showed no advantage over open pancreaticoduodenectomy (OPD) and more studies should focus on patient safety during learning curves [12].

Laparoscopic distal pancreatectomy was safe, while the complications various from different center [13]. As a minimally invasive and parenchyma-sparing procedure, laparoscopic central pancreatectomy (LCP) has been regarded historically as an alternative technique for benign or low-grade malignant tumors of the neck of the pancreas [14]. On the one hand, the short-term and long-term results of central pancreatectomy (CP) were controversial compared to distal pancreatectomy (DP) [15, 16]. On the other hand, LCP was challenge and had been rare reported worldwide [15].

Therefore, a high-volume center’s experience on laparoscopic pancreatectomy is still less and useful for the development and better understanding of this technique [8, 17, 18]. We have finished more than 500 LPDs and investigated the risk factors associated with complications and assessed the learning curves associated with LPD and vascular resection of LPD [11]. In this study, we will analyze the perioperative outcomes of all laparoscopic pancreatectomies for benign and low-grade malignant pancreatic tumors in a high-volume center in order to help facilitate a comprehensive and objective understanding of theses surgeries.

## Materials and methods

From February 2014 to February 2019, a total of 164 patients with benign or low-grade malignant pancreatic tumors were reviewed retrospectively in West China Hospital of Sichuan University. Twenty patients underwent laparoscopic enucleation were excluded from this study. Data were collected from medical record in terms of demographic characteristics (surgical procedure, age, sex, body mass index (BMI), American Society of Anesthesiology (ASA) score, comorbidities, history of abdominal surgery, hemoglobin, albumin, creatinine, tumor size, and pathological diagnosis), intra-operative and post-operative variables (conversion to open surgery, operative time, estimated blood loss, transfusion, post-operative hospital stay, and complications). Indication for surgery was based on a combination of patient symptoms and suspected pathology on radiological findings. For patients with recurrent pancreatitis, tumor size > 5 cm, high BMI or previous upper abdominal surgery, we adopted the policy of attempting laparoscopic surgery. For patients with tumors located at the neck of the pancreas, we will choose CP or DP and the final decision was made by the patient. Patients were grouped according to different surgical operations: laparoscopic pylorus-preserving pancreaticoduodenectomy (LPPPD), LCP and laparoscopic spleen-preserving distal pancreatectomy (LSPDP). Written consent was obtained from the patients associated in this study and this study was permitted by the Ethics Committee of West China Hospital, Sichuan University. The study was performed in accordance with the Declaration of Helsinki.

### Surgical techniques

**Fig. 1:**
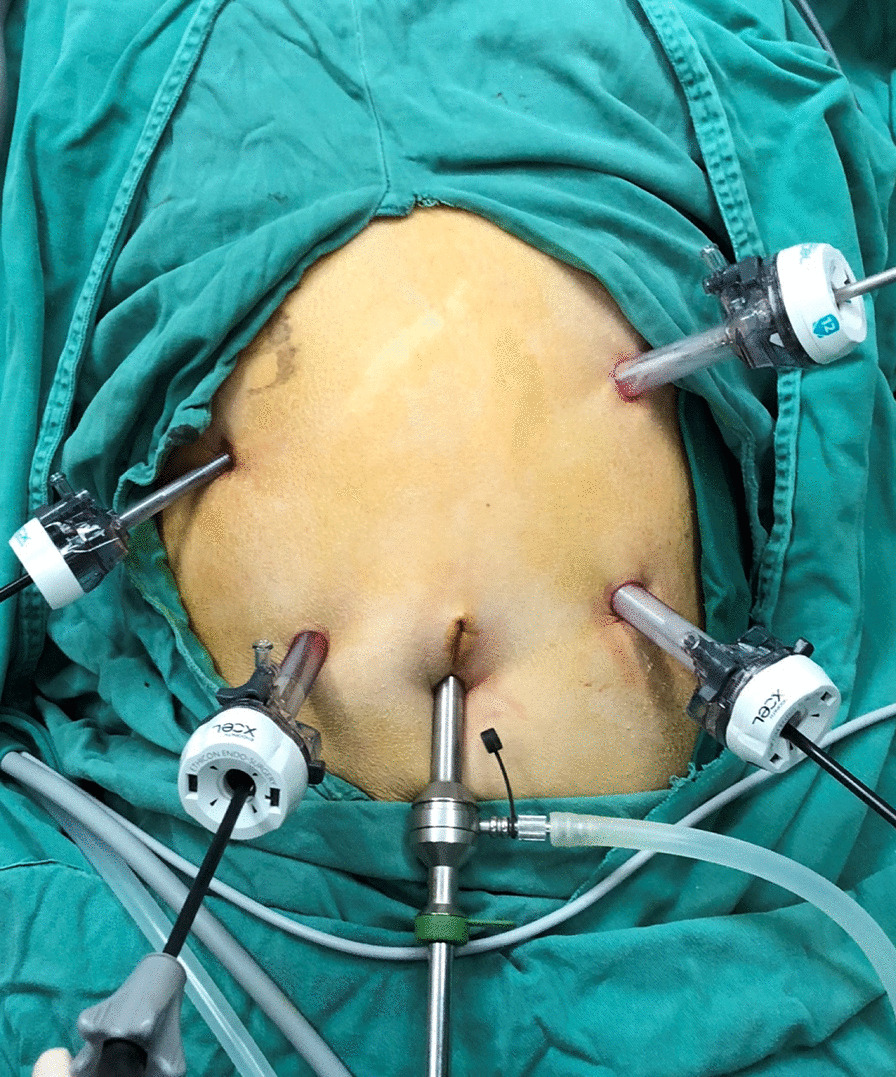
The routine distribution of five trocars

Under general anesthesia, the patient was placed in the supine position with legs apart. Five trocars were routinely needed. A 10-mm port was placed at the umbilicus for the telescope. After exploration, the other four trocars (three 12 mm, two 5 mm) were arranged in a V-shape (Fig. 1). Then open the gastrocolic ligament, procedures were started with tumor localization and staging. Finally, the specimen was removed via an enlarged trocar site and abdominal cavity were drained through other trocar sites.

#### LPPPD

**Fig. 2:**
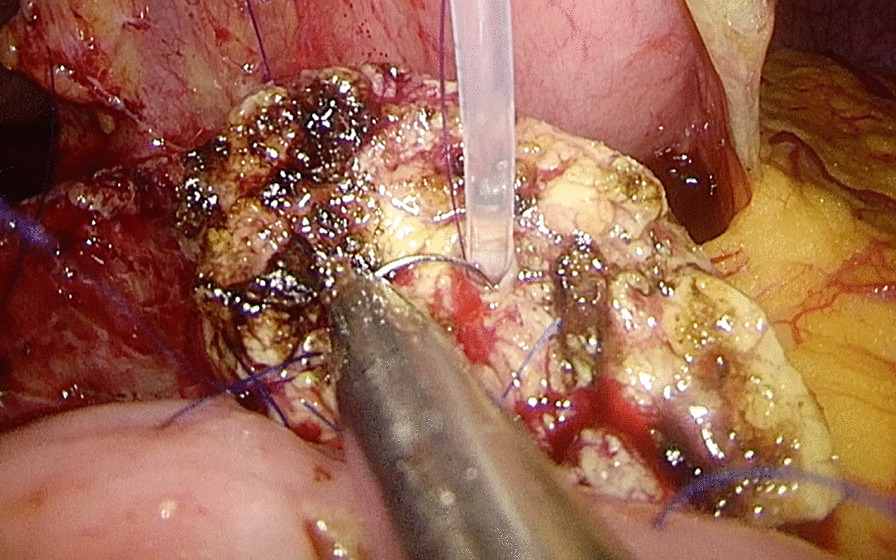
The second layer of Bing’s duct-to-mucosa manner, a figure-eight suture is performed between the posterior wall of the main pancreatic duct and the full layer of the jejunum

The LPPPD technique was described previously [19, 20]. Briefly, the hepatic flexure of the colon was fully taken down. Next, an extended Kocher maneuver was performed. Then, the duodenum, proximal jejunum, common hepatic duct, and pancreatic neck were dissected. Finally, pancreatojejunostomy was performed in Bing’s duct-to-mucosa manner [20] (Fig. 2) which has been described previously and hepaticojejunostomy was created as an end-to-side anastomosis.

#### LCP

The superior and inferior edges of the pancreas were mobilized to visualize the common hepatic artery (CHA), splenic artery (SA), superior mesenteric vein (SMV) and portal vein (PV). Laparoscopic ultrasonography was used to locate the tumor and mark the proximal and distal resection lines with a safety margin of 1–2 cm. Generally, a posterior pancreatic tunnel was created anterior to the PV. After transection the proximal pancreas with endoscopic linear stapler, the central part was carefully separated from the splenic vessels. The distal pancreas was transected with ultrasonic scalpel while the main pancreatic duct was transected with scissors. Finally, an end-to-side pancreaticojejunostomy (PJ) was also performed using the technique of Bing’s anastomosis which have been previously described [20].

#### LSPDP

**Fig. 3:**
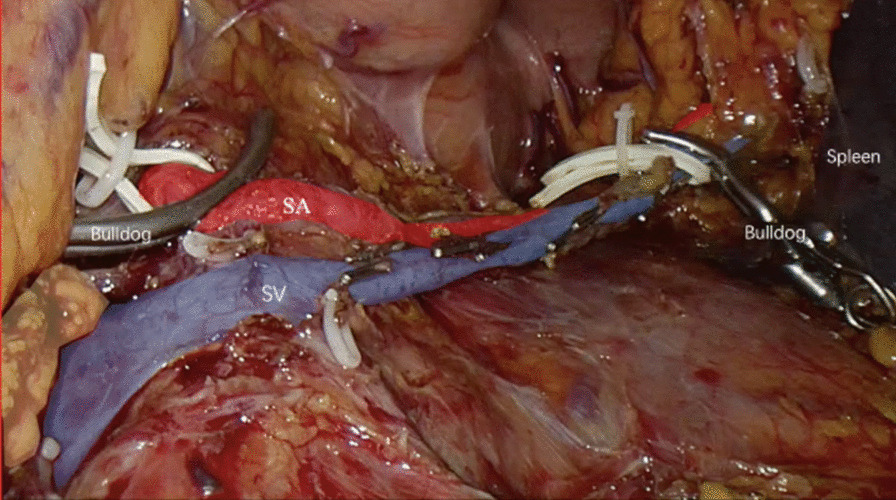
Dual-occlusion technique in laparoscopic spleen-preserving distal pancreatectomy: the proximal side of splenic artery and distal side of the splenic vein were occluded with laparoscopic bulldogs

The distal stomach was hanged with a rubber band which was retracted out of abdominal cavity from subxiphoid. Dissection was then continued at the inferior border of the pancreas in which the SMV was identified and a post-pancreatic neck tunnel was partially created. Then, the dissection was carried on at the superior border of pancreas in order to identify and isolate the CHA and SA and both were hanged with rubber band. The tail of the pancreas was mobilized from the splenic hilum until the splenic vein (SV) was identified and isolated. The patients underwent LSPDP in this study retained SA and SV according to the techniques described by Kimura. Pancreas was transected using linear stapler and then the proximal side of SA and distal side of SV were occluded with laparoscopic bulldogs (Fig. 3). Dissection was carried on from right to left between the tail of pancreas and splenic vessels using ultrasonic scalpel. Medium sized vessels were clipped and divided. Finally, the dissection was continued all the way to the splenic hilum.

##### *Definition*

Operation time was defined as the interval from the first skin incision to the final skin closure. Mortality was defined as death that occurred within 90 days of surgery. Postoperative complications such as postoperative pancreatic fistula (POPF), delayed gastric emptying (DGE), and postpancreatectomy hemorrhage (PPH) were defined according to the International Study Group of Pancreatic Surgery (ISGPS) [21–23], whereas severe complications were defined as Clavien–Dindo ≥ Grade 3 [24].

### Statistical analysis

The quantitative data results are expressed as mean (standard deviation) or median and interquartile range (IQR). The categorical data results are expressed as numbers and percentages of cases. Categorical variables were compared using the χ^2^ or Fisher exact test. Comparisons of continuous data were performed using the Student t test for normally distributed data; otherwise, the Mann–Whitney U test was used. All tests were two- tailed, and *P* < 0.05 was considered significant.

## Results

The demographic characteristics of the 144 patients were shown in Table 1. There were 53 male patients and 91 female patients in this study. There were 83 patients underwent LPPPD, 41 patients underwent LSPDP and 20 patients underwent LCP. The pylorus-preserving and spleen-preserving rate were 100%. The median age of these patients was 53.0 years (IQR 39.3–63.0 years). The median BMI was 21.5 kg/m^2^ (IQR 19.7–24.0 kg/m^2^). The pathologic diagnosis of these patients included 24 (16.7%) cases of solid pseudopapillary tumors, 39 (27.1%) cases of pancreatic intraductal papillary mucinous tumors, 29 (20.1%) cases of pancreatic neuroendocrine tumors, 18 (12.5%) cases of serous cystadenoma and 34 (23.6%) cases of mucinous cystadenoma. The median diameter of tumor was 3.4 cm (IQR 2.8–4.0 cm).

**Table 1:** The demographic characteristics of all patients

Variables	Mean ± SD or median (IQR) or case number
Cases (Total)	144
LPPPD	83 (57.6%)
LSPDP	41 (28.5%)
LCP	20 (13.9%)
Sex (male/female)	53/91
Age (years, IQR)	53.0 (39.3–63.0)
BMI (Kg/m^2^, IQR)	21.5 (19.7–24.0)
ASA score	
II (n, %)	134 (93.1%)
III (n, %)	10 (6.9%)
Comorbidities* (n, %)	36 (25.0%)
History of abdominal surgery^†^ (n, %)	27 (18.8%)
Hemoglobin (g/L, mean ± SD)	126.2 ± 16.8
Albumin (g/L, mean ± SD)	41.8 ± 3.7
Creatinine (μmol/L, IQR)	87.0 (69.0–98.0)
Pathological diagnosis	
Intraductal papillary mucinous neoplasm^‡^	39 (27.1%)
Solid pseudopapillary tumor	24 (16.7%)
Cystadenoma (mucinous/serous)	52 (34/18, 36.1%)
Neuroendocrine tumor^§^	29 (20.1%)

*SD* standard deviation, *IQR* interquartile range, *LPPPD* laparoscopic pylorus-preserving pancreaticoduodenectomy, *LSPDP* laparoscopic spleen-preserving distal pancreatectomy, *LCP* laparoscopic central pancreatectomy, *BMI* body mass index, *ASA* score American Society of Anesthesiologists classification score

*Comorbidities including chronic obstructive pulmonary disease, hypertension, diabetes and cardiovascular disease

^†^History of abdominal surgery including cholecystectomy, appendectomy, biliary tract and gastric surgery

^‡^Only including low-grade and moderate-grade dysplasia

^§^Only including G1 and G2

The surgical outcomes and operative details of these patients were shown in Table 2. One patient (0.7%) converted to open pancreaticoduodenectomy due to uncontrolled bleeding from first jejunal vein. The median operative time was 230 min (IQR 190–298.8 min). The median estimated blood loss was 100 ml (range 20–1000 ml). Only two patients in LPPPD group require blood transfusion. The median postoperative hospital stay was 10 days (IQR 9–14 days). The postoperative severe complication was 4.2%. No patients suffered from bile leakage. Fourteen patients (9.7%) suffered from pancreatic fistula (grade B, 14 cases; grade C 0 case). Nine patients (6.3%) suffered from delayed gastric emptying and was cured by conservative therapy. Two patients (1.4%) suffered from PPH and one was cured by interventional embolization and the other patient need reoperation. The 90-days mortality was 0.

**Table 2:** The surgical outcomes and postoperative details of all patients

Variables	Median (IQR) or case number
No. of patients	144
Tumor size (cm, IQR)	3.4 (2.8–4.0)
Operative time (min)	230.0 (190.0–298.8)
Estimated blood loss (ml)	100.0 (20.0–1000.0)^†^
Conversion to open surgery (n, %)	1 (0.7%)
Blood transfusion (n, %)	2 (1.4%)
Complications (Clavien-Dindo ≥ Grade 3)	6 (4.2%)
Chylous leakage	1 (0.7%)
Bile leakage	0 (0%)
Pancreatic fistula (n, %)	
Biochemical leak	35 (24.3%)
Grade B	14 (9.7%)
Grade C	0 (0%)
PPH (n, %)	2 (1.4%)
DGE	9 (6.3%)
Reoperation* (n, %)	1 (0.7%)
90-Day mortality (n, %)	0 (0%)
Postoperative hospital stay (days)	10 (9–14)

*PPH* post-pancreatectomy hemorrhage, *DGE* delayed gastric emptying

*Reoperation: only including open surgery, laparoscopic surgery and interventional surgery

^†^Data was indicated with median and range

The comparison of demographic characteristics among three groups were shown in Table 3. Comparing with patients in LCP group, the patients in LPPPD group and LSPDP group had lower BMI (21.5 ± 3.2 vs. 24.0 ± 3.7 kg/m^2^, *P* = 0.004 and 22.0 ± 2.7 vs. 24.0 ± 3.7, *P* = 0.024, respectively). The patients in LPPPD group had lower level of hemoglobin (124.2 ± 19.4 vs.131.8 ± 7.6 g/L, *P* = 0.048). The rest variables were comparable among three groups (all *P* > 0.05).

**Table 3:** The demographic characteristics of three groups

Variables	LPPPD n = 83	LCP n = 20	LSPDP n = 41	*P value*	
				*P*_*1*_	*P*_*2*_
Age (years)	54.8 ± 13.4	48.8 ± 15.7	45.3 ± 14.4	0.084	0.466
Gender (male/female)	36/47	7/13	10/31	0.495	0.386
BMI (kg/m^2^)	21.5 ± 3.2	24.0 ± 3.7	22.0 ± 2.7	**0.004**	**0.024**
ASA score					
II (n, %)	80 (96.4%)	17 (85.0%)	37 (90.2%)	0.086	0.674
III (n, %)	3 (3.6%)	3 (15.0%)	4 (9.8%)		
Comorbidities* (n, %)	16 (19.3%)	7 (35.0%)	13 (31.7%)	0.224	0.797
History of abdominal surgery^†^	13 (15.7%)	4 (20.0%)	10 (24.4%)	0.894	0.953
Hemoglobin (g/L)	124.2 ± 19.4	131.8 ± 7.6	127.5 ± 11.1	**0.048**	0.192
Albumin (g/L)	41.9 ± 3.7	42.5 ± 3.4	41.3 ± 4.1	0.484	0.229
Creatinine (μmol/L)	87.0 (72.0–98.0)	83.0 (57.5–98.8)	87.0 (67.0–98.5)	0.496	0.585

Bold value indicates statistical significance (*P* < 0.05; *P*_1_: LPPPD group vs. LCP group; *P*_2_: LSPDP group vs. LCP group)

*LPPPD* laparoscopic pylorus-preserving pancreaticoduodenectomy, *LSPDP* laparoscopic spleen-preserving distal pancreatectomy, *LCP* laparoscopic central pancreatectomy, *BMI* body mass index, *ASA* score American Society of Anesthesiologists classification score

*Comorbidities including chronic obstructive pulmonary disease, hypertension, diabetes and cardiovascular disease

^†^History of abdominal surgery including cholecystectomy, appendectomy, biliary tract and gastric surgery

The comparison of surgical outcomes and postoperative details among three groups were shown in Table 4. Comparing with LCP group, the LPPPD group had longer operation time (300.4 ± 89.7 vs. 197.5 ± 30.5 min, *P* < 0.001) while LSPDP group had shorter operation time (174.8 ± 46.4 vs. 197.5 ± 30.5 min, *P* = 0.027). Compare with LCP group, the LPPPD and LSPDP group had more blood loss [140.0 (50.0–1000.0) vs. 50.0 (20.0–200.0) ml *P* < 0.001 and 100.0 (20.0–300.0) vs. 50.0 (20.0–200.0 ml, *P* = 0.039, respectively], shorter postoperative hospital stays [11.0 (9.0–14.0) vs. 14.5 (12.3–23.0) d, *P* < 0.001 and 10.0 (9.0–11.0) vs. 14.5 (12.3–23.0), *P* < 0.001, respectively], lower rate of clinically relevant POPF [3 (3.6%) vs. 8 (40.0%), *P* < 0.001 and 3 (7.3%) vs. 8 (40.0%), *P* = 0.006, respectively], lower rate of PPH [0 (0%) vs. 2 (10.0%), *P* = 0.036 and (0%) vs. 2 (10.0%) *P* = 0.104, respectively] and lower rate of postoperative severe complications [2 (2.4%) vs. 4 (20.0%), *P* = 0.012 and 0 (0%) vs. 4 (20.0%), *P* = 0.009, respectively]. Two patients in LCP groups suffered PPH, one of them was found a vascular clamp falling off 8 h after the operation and the vascular stump was ligated by reoperation and the other patient suffered from abdominal pain and intraperitoneal hemorrhage 9 days after discharge, which was found to be a small branch of pancreaticoduodenal artery by angiography and cured by interventional embolization.

**Table 4:** The outcomes and postoperative details of three groups

Variables	LPPPD n = 83	LCP n = 20	LSPDP n = 41	*P* value	
				*P*_1_	*P*_2_
Tumor size (cm)	3.8 ± 1.1	2.8 ± 0.8	3.6 ± 2.2	**< 0.001**	0.060
OT (min)	300.4 ± 89.7	197.5 ± 30.5	174.8 ± 46.4	**< 0.001**	**0.027**
Conversion (n, %)	1 (1.2%)	0 (0%)	0 (0%)	1.000	NA^†^
EBL (mL)	140.0 (50.0–1000.0)	50.0 (20.0–200.0)	100.0 (20.0–300.0)	**< 0.001**	**0.039**
BT (n, %)	2 (2.4%)	0 (0%)	0 (0%)	1.000	NA^†^
POHS (days)	11.0 (9.0–14.0)	14.5 (12.3–23.0)	10.0 (9.0–11.0)	**< 0.001**	**< 0.001**
CR-POPF (n, %)	3 (3.6%)	8 (40.0%)	3 (7.3%)	**< 0.001**	**0.006**
PPH (n, %)	0 (0%)	2 (10.0%)	0 (0%)	**0.036**	0.104
DGE (n, %)	5 (6.0%)	3 (15.0%)	1 (2.4%)	0.378	0.090
Reoperation*	0 (0%)	1 (5.0%)	0 (0%)	0.194	0.328
CL (n, %)	1 (1.2%)	0 (0%)	0 (0%)	1.000	NA^†^
Biliary fistula	0 (0%)	0 (0%)	0 (0%)	NA^†^	NA^†^
Complications **(Clavien—Dindo ≥ Grade 3)**	2 (2.4%)	4 (20.0%)	0 (0%)	**0.012**	**0.009**
90-Day mortality	0 (0.0%)	0 (0%)	0 (0%)	NA^†^	NA^†^
Follow-up					
Postoperative pancreatitis	5 (6.0%)	2 (10.0%)	0 (0%)	0.889	0.104
Pancreatin treatment	33 (39.8%)	3 (15.0%)	10 (24.4%)	**0.037**	0.390
Insulin treatment	15 (18.1%)	0 (0%)	5 (12.2%)	**0.040**	**0.041**

Bold value indicates statistical significance (*P* < 0.05; *P*_1_: LPPPD group vs. LCP group; *P*_2_: LSPDP group vs. LCP group)

*LPPPD* laparoscopic pylorus-preserving pancreaticoduodenectomy, *LSPDP* laparoscopic spleen-preserving distal pancreatectomy, *LCP* laparoscopic central Pancreatectomy, *OT* operative time, *EBL* estimated blood loss, *BT* blood transfusion, *POHS* post-operative hospital stay, *CR-POPF* clinically relevant postoperative pancreatic fistula, *PPH* post-pancreatectomy hemorrhage, *DGE* delayed gastric emptying, *CL* Chylous leakage

*Reoperation: only including open surgery, laparoscopic surgery and interventional surgery

^†^NA: Not Applicable

The patients were followed up by telephone interviews. Five patients in LPPPD group and 2 patients in LCP group developed postoperative pancreatitis after surgery. There were no significant differences (*P* = 0.889). LCP group had lower proportion of postoperative pancreatin and insulin treatment compared to LPPPD group and LSPDP group (pancreatin: 15% vs. 39.8%, *P* = 0.037, 15% vs. 24.4%, *P* = 0.390; insulin: 0 vs. 18.1%, *P* = 0.040, 0 vs. 12.2%, *P* = 0.041) (Table 4).

## Discussion

In this study, we found laparoscopic pancreatectomy could be safely performed for benign and low-grade malignant pancreatic tumors in a high-volume center as the postoperative severe complication was 4.2%, the 90-days mortality was 0 and the conversion rate was 0.7%. In our center, we usually do pylorus-preserving pancreatoduodenectomy and spleen-preserving distal pancreatectomy for benign and low-grade malignant pancreatic tumors and the preserving rates were 100% in our report. However, organ-preserving surgery were controversial and more researches were need to prove the advantages [25, 26].

Then we carried out a subgroup analysis based on different surgical operations. Although LPPPD, which has the longest operation time and highest intraoperative blood loss as shown in Table 4, was considered to be the most complex operation compared with LCP and LSPDP [27]. LPPPD has the lowest incidence of clinically relevant POPF (3.6%). This may because we use the technique of Bing’s duct-to-mucosa manner for pancreatojejunostomy which could reduce the incidence of clinically relevant POPF [20].

LCP is an alternative technique for benign or low-grade malignant tumors of the neck of the pancreas. Most studies agreed that CP was superior to DP regarding the preservation of pancreatic function [28]. While Lee’s [16] report came up with different opinions through an interesting study using pancreatic volumetry to evaluation of pancreatic exocrine function. This may because most patients (81.8%) underwent pancreaticogastrostomy (PG) in Lee’s report which could affect the function of pancreas [16, 29]. We believes as parenchyma-sparing procedure, LCP can preserve long-term endocrine and exocrine functions, which could be more important for patients’ quality of life compared with LPPPD and LSPDP [30]. However, a major drawback is that two transected surfaces of the pancreas remain after LCP, thereby exposing patients to an increased risk of POPF [15]. At present, the major approaches to pancreatic reconstruction are oversewing of the cephalic pancreatic stump and pancreatic anastomosis to the distal stump [31]. The latter approach is technically challenging in LCP because of the softness of the pancreas tissue and the small size of the pancreatic duct, which may also increase the risk of leakage [31, 32]. Despite advances in surgical techniques and perioperative management, the rate of postoperative POPF remains as high as 40–70% [33–35]. In our report, the rate of POPF was 40% which was comparable to the literature while much higher than that in LPPPD and LSPDP.

Postoperative hemorrhage is another sever complication after pancreatectomy as it is associated with a high mortality. Only two (1.4%) patients in the LCP groups had PPH, of which 1 (0.7%) patient met the diagnosed of late PPH according to the ISGPS definition (i.e., occurring > 24 h after pancreatic resection) [22] We have a lower rate of PPH compare to the literature which the incidence of late PPH ranged from 3 to 16% [36]. The main reason for late PPH was POPF, as we have a low rate of POPF and we also adopt covering of GDA stumps and CHA with the round ligament to prevent PPH after LPPPD. Gastroduodenal artery stump (GDA) was the most frequent origin of the hemorrhage, followed by the CHA and SA [36]. Interventional angiography, which was also successful in our report, appears to be associated to lower mortality as compared to relaparotomy and endoscopy as first intervention for late PPH [36].

Our study has several limitations, including lack of open data comparison and long-term outcomes. Nonetheless, these cases were consecutive patients from a single high-volume institution, thereby possibly eliminating some potential biases. Finally, the safety and the feasibility of this technique should be verified by additional prospective randomized controlled trials at different institutions.

## Conclusion

Overall, laparoscopic pancreatectomy could be safely performed for benign and low-grade malignant pancreatic tumors while the decision to perform laparoscopic central pancreatectomy should be made carefully for fit patients who can sustain a significant postoperative morbidity and could benefit from the excellent long-term results even in a high-volume center.

## Data Availability

The datasets used and analyzed during the current study available from the corresponding author on reasonable request.

## Abbreviations

LPPPD: Laparoscopic pylorus-preserving pancreaticoduodenectomy
LSPDP: Laparoscopic spleen-preserving distal pancreatectomy
LCP: Laparoscopic central pancreatectomy
CP: Central pancreatectomy
DP: Distal pancreatectomy
ASA: American Society of Anesthesiology
TB: Total bilirubin
CHA: Common hepatic artery
SA: Splenic artery
SMV: Superior mesenteric vein
PJ: Pancreaticojejunostomy
SV: Splenic vein
POPF: Postoperative pancreatic fistula
DGE: Delayed gastric emptying
PPH: Postpancreatectomy hemorrhage
ISGPS: International Study Group of Pancreatic Surgery
GDA: Gastroduodenal artery stump

## References

[1] Gagner M, Pomp A, Herrera MF (1996). Early experience with laparoscopic resections of islet cell tumors. Surgery.

[2] Poves I, Burdio F, Morato O, Iglesias M, Radosevic A, Ilzarbe L, Visa L, Grande L (2018). Comparison of perioperative outcomes between laparoscopic and open approach for pancreatoduodenectomy: the PADULAP randomized controlled trial. Ann Surg.

[3] de Rooij T, van Hilst J, van Santvoort H, Boerma D, van den Boezem P, Daams F, van Dam R, Dejong C, van Duyn E, Dijkgraaf M (2019). Minimally invasive versus open distal pancreatectomy (LEOPARD): a multicenter patient-blinded randomized controlled trial. Ann Surg.

[4] Nassour I, Wang SC, Christie A, Augustine MM, Porembka MR, Yopp AC, Choti MA, Mansour JC, Xie XJ, Polanco PM (2018). Minimally invasive versus open pancreaticoduodenectomy: a propensity-matched study from a national cohort of patients. Ann Surg.

[5] Palanivelu C, Senthilnathan P, Sabnis SC, Babu NS, Srivatsan Gurumurthy S, Anand Vijai N, Nalankilli VP, Praveen Raj P, Parthasarathy R, Rajapandian S (2017). Randomized clinical trial of laparoscopic versus open pancreatoduodenectomy for periampullary tumours. Br J Surg.

[6] Edwin B, Sahakyan MA, Hilal MA, Besselink MG, Braga M, Fabre J-M, Fernández-Cruz L, Gayet B, Kim SC, Khatkov IE (2017). Laparoscopic surgery for pancreatic neoplasms: the European association for endoscopic surgery clinical consensus conference. Surg Endosc.

[7] Mehrabi A, Hafezi M, Arvin J, Esmaeilzadeh M, Garoussi C, Emami G, Kossler-Ebs J, Muller-Stich BP, Buchler MW, Hackert T (2015). A systematic review and meta-analysis of laparoscopic versus open distal pancreatectomy for benign and malignant lesions of the pancreas: it's time to randomize. Surgery.

[8] Rosok BI, Marangos IP, Kazaryan AM, Rosseland AR, Buanes T, Mathisen O, Edwin B (2010). Single-centre experience of laparoscopic pancreatic surgery. Br J Surg.

[9] van Hilst J, de Rooij T, Bosscha K, Brinkman DJ, van Dieren S, Dijkgraaf MG, Gerhards MF, de Hingh IH, Karsten TM, Lips DJ (2019). Laparoscopic versus open pancreatoduodenectomy for pancreatic or periampullary tumours (LEOPARD-2): a multicentre, patient-blinded, randomised controlled phase 2/3 trial. Lancet Gastroenterol Hepatol.

[10] Adam MA, Thomas S, Youngwirth L, Pappas T, Roman SA, Sosa JA (2017). Defining a hospital volume threshold for minimally invasive pancreaticoduodenectomy in the United States. JAMA Surg.

[11] Wang X, Cai Y, Jiang J, Peng B (2020). Laparoscopic pancreaticoduodenectomy: outcomes and experience of 550 patients in a single institution. Ann Surg Oncol.

[12] Nickel F, Haney CM, Kowalewski KF, Probst P, Limen EF, Kalkum E, Diener MK, Strobel O, Muller-Stich BP, Hackert T (2020). Laparoscopic versus open pancreaticoduodenectomy: a systematic review and meta-analysis of randomized controlled trials. Ann Surg.

[13] Eguia E, Kuo PC, Sweigert P, Nelson M, Aranha GV, Abood G, Godellas CV, Baker MS (2019). The laparoscopic approach to distal pancreatectomy is a value-added proposition for patients undergoing care in moderate-volume and high-volume centers. Surgery.

[14] Rotellar F, Pardo F (2010). Laparoscopic middle pancreatectomy minimizes the procedure and maximizes the benefit. Surgery.

[15] Song KB, Kim SC, Park KM, Hwang DW, Lee JH, Lee DJ, Lee JW, Jun ES, Shin SH, Kim HE (2015). Laparoscopic central pancreatectomy for benign or low-grade malignant lesions in the pancreatic neck and proximal body. Surg Endosc.

[16] Lee DH, Han Y, Byun Y, Kim H, Kwon W, Jang JY (2020). Central pancreatectomy versus distal pancreatectomy and pancreaticoduodenectomy for benign and low-grade malignant neoplasms: a retrospective and propensity score-matched study with long-term functional outcomes and pancreas volumetry. Ann Surg Oncol.

[17] Song KB, Kim SC, Park JB, Kim YH, Jung YS, Kim MH, Lee SK, Seo DW, Lee SS, Park DH (2011). Single-center experience of laparoscopic left pancreatic resection in 359 consecutive patients: changing the surgical paradigm of left pancreatic resection. Surg Endosc.

[18] Song KB, Kim SC, Lee W, Hwang DW, Lee JH, Kwon J, Park Y, Lee SJ, Park G (2020). Laparoscopic pancreaticoduodenectomy for periampullary tumors: lessons learned from 500 consecutive patients in a single center. Surg Endosc.

[19] Cai Y, Chen S, Peng B (2019). Two-surgeon model in laparoscopic pancreaticoduodenectomy. Surg Laparosc Endosc Percutan Tech.

[20] Cai Y, Luo H, Li Y, Gao P, Peng B (2019). A novel technique of pancreaticojejunostomy for laparoscopic pancreaticoduodenectomy. Surg Endosc.

[21] Wente MN, Bassi C, Dervenis C, Fingerhut A, Gouma DJ, Izbicki JR, Neoptolemos JP, Padbury RT, Sarr MG, Traverso LW (2007). Delayed gastric emptying (DGE) after pancreatic surgery: a suggested definition by the International Study Group of Pancreatic Surgery (ISGPS). Surgery.

[22] Wente MN, Veit JA, Bassi C, Dervenis C, Fingerhut A, Gouma DJ, Izbicki JR, Neoptolemos JP, Padbury RT, Sarr MG (2007). Postpancreatectomy hemorrhage (PPH)–an International Study Group of Pancreatic Surgery (ISGPS) definition. Surgery.

[23] Bassi C, Marchegiani G, Dervenis C, Sarr M, Abu Hilal M, Adham M, Allen P, Andersson R, Asbun HJ, Besselink MG (2017). The 2016 update of the International Study Group (ISGPS) definition and grading of postoperative pancreatic fistula: 11 years after. Surgery.

[24] Dindo D, Demartines N, Clavien PA (2004). Classification of surgical complications: a new proposal with evaluation in a cohort of 6336 patients and results of a survey. Ann Surg.

[25] Klaiber U, Probst P, Strobel O, Michalski CW, Dörr-Harim C, Diener MK, Büchler MW, Hackert T (2018). Meta-analysis of delayed gastric emptying after pylorus-preserving versus pylorus-resecting pancreatoduodenectomy. Br J Surg.

[26] Panda N, Bansal NK, Narsimhan M, Ardhanari R, Bronson JRB (2015). Spleen-preserving versus spleen-sacrificing distal pancreatectomy in laparoscopy and open method-perioperative outcome analysis—14 years experience. Indian J Surg.

[27] Tan HL, Syn N, Goh BKP (2019). Systematic review and meta-analysis of minimally invasive pancreatectomies for solid pseudopapillary neoplasms of the pancreas. Pancreas.

[28] Dragomir MP, Sabo AA, Petrescu GED, Li Y, Dumitrascu T (2019). Central pancreatectomy: a comprehensive, up-to-date meta-analysis. Langenbecks Arch Surg.

[29] Benini L, Gabbrielli A, Cristofori C, Amodio A, Butturini G, Cardobi N, Sozzi C, Frulloni L, Mucelli RP, Crino S (2019). Residual pancreatic function after pancreaticoduodenectomy is better preserved with pancreaticojejunostomy than pancreaticogastrostomy: a long-term analysis. Pancreatology.

[30] Xiao W, Zhu J, Peng L, Hong L, Sun G, Li Y (2018). The role of central pancreatectomy in pancreatic surgery: a systematic review and meta-analysis. HPB (Oxford).

[31] Iacono C, Verlato G, Ruzzenente A, Campagnaro T, Bacchelli C, Valdegamberi A, Bortolasi L, Guglielmi A (2013). Systematic review of central pancreatectomy and meta-analysis of central versus distal pancreatectomy. Br J Surg.

[32] Sperti C, Beltrame V, Milanetto AC, Moro M, Pedrazzoli S (2010). Parenchyma-sparing pancreatectomies for benign or border-line tumors of the pancreas. World J Gastrointest Oncol.

[33] Goudard Y, Gaujoux S, Dokmak S, Cros J, Couvelard A, Palazzo M, Ronot M, Vullierme MP, Ruszniewski P, Belghiti J (2014). Reappraisal of central pancreatectomy a 12-year single-center experience. JAMA Surg.

[34] Gonzalez F, Mesleh MG, Lukens FJ, Wallace MB, Asbun HJ, Stauffer JA (2013). Laparoscopic central pancreatectomy and pancreaticogastrostomy for the management of a proximally migrated pancreatic stent. JOP: J Pancreas.

[35] Paiella S, De Pastena M, Faustini F, Landoni L, Pollini T, Bonamini D, Giuliani T, Bassi C, Esposito A, Tuveri M (2019). Central pancreatectomy for benign or low-grade malignant pancreatic lesions—a single-center retrospective analysis of 116 cases. Eur J Surg Oncol.

[36] Floortje van Oosten A, Smits FJ, van den Heuvel DAF, van Santvoort HC, Molenaar IQ (2019). Diagnosis and management of postpancreatectomy hemorrhage: a systematic review and meta-analysis. HPB.

